# Investigating the impact of terrorist attacks on the mental health of emergency responders: systematic review

**DOI:** 10.1192/bjo.2022.69

**Published:** 2022-06-03

**Authors:** Ulrich Wesemann, Briana Applewhite, Hubertus Himmerich

**Affiliations:** Department of Psychiatry, Psychotherapy and Psychotraumatology, Bundeswehr Hospital Berlin, Germany; Department of Psychological Medicine, Institute of Psychiatry, Psychology and Neuroscience, King's College London, UK; Department of Psychological Medicine, Institute of Psychiatry, Psychology and Neuroscience, King's College London, UK

**Keywords:** Systematic review, terrorist attack, mental health, post-traumatic stress disorder, emergency service personnel

## Abstract

**Background:**

Terrorist attacks have strong psychological effects on rescue workers, and there is a demand for effective and targeted interventions.

**Aims:**

The present systematic review aims to examine the mental health outcomes of exposed emergency service personnel over time, and to identify risk and resilience factors.

**Method:**

A literature search was carried out on PubMed and PubPsych until 27 August 2021. Only studies with a real reported incident were included. The evaluation of the study quality was based on the Quality Assessment Tool for Quantitative Studies, and the synthesis used the ‘Guidance on the Conduct of Narrative Synthesis in Systematic Reviews’.

**Results:**

Thirty-three articles including 159 621 individuals were identified, relating to five different incidents with a post-event time frame ranging from 2 weeks to 13 years. The post-traumatic stress disorder prevalence rates were between 1.3 and 16.5%, major depression rates were between 1.3 and 25.8%, and rates for specific anxiety disorders were between 0.7 and 14%. The highest prevalence rates were found after the World Trade Center attacks. Reported risk factors were gender, no emergency service training, peritraumatic dissociation, spatial proximity to the event and social isolation.

**Conclusions:**

The inconsistency of the prevalence rates may be attributable to the different severities of the incidents. Identified risk factors could be used to optimise training for emergency personnel before and after catastrophic events. Voluntary repetitive screening of rescue workers for mental health symptoms is recommended.

## Introduction

### Definitions

Terrorist attacks can be defined by their intent to intimidate a larger population more so than the immediate victims. The action must be outside the context of legitimate warfare activities and directed toward achieving a political, economic, religious or social goal.^1^ According to the Global Terrorism Database, over the past 10 years, explosives have been the most commonly used method of terrorist attack, followed by firearms, incendiary weapons and melee weapons.^1^ The type of attack also has an impact on the tasks of the emergency services on site. This is relevant for mission preparation, as well as for their own personal safety and risk assessment.^2^

The professional groups deployed usually include firefighters, police officers and rescue services, which are supplemented by other security forces, depending on the type of attack and the country affected.^3^ The psychological consequences of terrorist attacks are more severe than those of natural disasters for emergency services personnel and the general population.^4^ In addition to the personal involvement and event itself, evaluation processes play an important role in mental processing. This explains why the civilian population directly affected is the most heavily burdened, followed by the deployed emergency services personnel and then the general population.^5^

### Occupational and gender differences

Various studies have shown that emergency services personnel from different professions also react different emotionally to a major attack.^6–8^ A recently published systematic review shows that police officers have a higher resilience to post-traumatic stress disorder (PTSD) and post-traumatic stress symptoms (PTSS) because of their training.^9^

Studies on gender differences indicate a higher burden on female emergency services personnel.^10–13^ Regional differences were also identified. A meta-analysis reported higher prevalence rates of PTSD in Western Europe compared with North America.^9^ In contrast, another systematic review found that studies from Asia have a higher prevalence of PTSD than studies from Europe, but a lower prevalence than studies from North America.^14^ These differences and contradictions support the above-mentioned hypothesis that there are other relevant factors that play a role in mental processing besides the terrorist event.

### Risk factors and crisis intervention

Resilience, psychiatric history and current mental health are some of these identified risk factors.^15–19^ The physical proximity to the critical event has also been shown to influence later symptoms, such as mood, anxiety or sleep disorders.^20,21^ This underscores that the spectrum of symptoms is much broader than that of PTSD, ranging from sleep disorders to cognitive, emotional, physical and behavioural problems, to substance use disorders.^22–28^

After the terrorist attack at Berlin's Breidtscheidpatz, it became clear that there was an urgent need for new crisis intervention measures. These contemporary measures must also be able to be carried out by semi-professional providers and must not contain psychotherapeutic or pharmacotherapeutic elements exclusively.^29^ To develop specific prevention tools, it is important to record and assess the range of mental health symptoms following critical incidents.^3^ Risk and resilience factors must also be taken into account.

### Purpose

The purpose of this review is to provide information about the psychological impact on emergency services personnel involved in intentionally induced high-stress and traumatic situations. The following questions are of special interest:

- What psychological effects can be expected on rescue workers after terrorist attacks?
- Are there gender or occupational differences in mental health outcomes after terrorist attacks?
- How do rescue workers progress psychologically after a terrorist attack?

After reviewing the search results, the following question was added:

- Which risk and resilience factors affect mental health in this context?

## Method

This review was designed in accordance with the Preferred Reporting Items for Systematic Reviews and Meta-Analyses (PRISMA) protocol for systematic reviews^30^ and registered with the International Prospective Register of Systematic Reviews (PROSPERO; identification number CRD42021241230). Ethics approval and informed consent were not required for this systematic review.

### Search strategy

A PubMed and PubPsych search was carried out with the following search terms up to 7 February 2021: ((((terrorist attack) OR (amok[Title/Abstract]))) AND (((mental health[Title/Abstract]) OR (ptsd[Title/Abstract])) OR (stress[Title/Abstract]))) AND (((((police[Title/Abstract]) OR (firefighter[Title/Abstract])) OR (first line[Title/Abstract])) OR (emergency responder[Title/Abstract])) OR (service personnel[Title/Abstract])). A second search took place on 27 August 2021. The publication date was unlimited, and only publications in English were taken into account.

### Eligibility

The titles and abstracts of the identified articles were then checked for inclusion criteria. Only peer-reviewed studies with a real terroristic or amok incident were included, as well as those with an examination of the mental health of the emergency responders. Trials or simulations of terroristic events were excluded. Both cross-sectional and longitudinal studies with their own data collection were included; reviews and meta-analyses without their own data collection were excluded. The positively screened articles were then included in the qualitative analysis.

### Data extraction

The data synthesis was divided into four different groups: emergency service personnel, gender, symptoms and time after the event. The literature was evaluated according to the PICO scheme (patient/population, intervention, comparison and outcomes) for reviews. The professional groups (P) included police officers, firefighters, rescue services, non-profit organisations (NGOs) and providers of psychosocial services. The exposure group (I) were professional first responders deployed during or after a terrorist attack or rampage. Since these incidents are examined almost exclusively within a naturalistic study design, the comparison group (C) was defined as emergency personnel from the same occupational groups that were not used for the incident. Mental health effects of the emergency service personnel were defined as the result (O). The effect measures of the main results are prevalence rates (in percent) and odds ratios, if applicable. One researcher (U.W.) conducted the literature search and data extraction. A second researcher (H.H.) conducted the literature search independently, and disagreements were resolved with the consent of both. All emergency services personnel related mental health outcomes were extracted. Data compared with control groups other than those specified above were not taken into account in the results. No special software was used for the review. The result data were descriptively collected in Microsoft Excel for Windows 10. Table 1 gives an overview of the literature research and the integration into the PICO scheme.

**Table 1:** Characteristics of the included studies that report on the mental health of emergency responders after terrorist attacks

Author	Sample size, occupational group and demographics	Study design and time after incident	Research methods	Outcome	Comments
Biggs et al, 2010^31^	90 disaster workers, 75.6% male, mean age 35.6 years, 83.3% White, 77.7% college degree or higher, 56.7% married	Cross-sectional cohort study without control group; 2–3 weeks after the 9/11 WTC attacks	Survey and questionnaires: Zung Self-Rating Depression Scale; SF-8 Health Survey; THQ; PDEQ	ASD (questionnaire): 14.6%; depression (questionnaire): 25.8%; high impairment: 33.3%; one or more symptoms of peritraumatic dissociation: 83.3%	Occupational groups not specified further, 10.9% with comorbid depression and ASD; tobacco users: 37.9–33.3% with ASD
Bowler et al, 2010^32^	4017 police officers, 85% male, 70.4% White, 99% high school or higher, 67.9% married	Cross-sectional study without control group; 2–3 years after the 9/11 WTC attacks	Questionnaires: PCL	PTSD: 7.8%; gender differences in PTSD: women 13.9%, men 7.4%	General risk factors: event related injury, older age; for men: proximity to event, Hispanic ethnicity; for women: witnessing horror, education less than college degree
Bowler et al, 2012^33^	2940 police officers, 86.0% male, 73.9% White, 99% high school or higher, 74.1% married	Longitudinal study without control group; 2–3 years and 5–6 years after the 9/11 WTC attacks	Questionnaires: PCL	PTSD increased significantly to 16.5%	PTSD: no more gender-specific differences in wave 2, but still more intrusive symptoms in women; general: emotionally numb 6.7% in wave 1 to 15.6% in wave 2
Bowler et al, 2016^34^	1884 police officers, 76.9% male, mean age 37.9 years, Hispanic ethnicity 14.1%	Longitudinal study without control group; 2–3, 5–6 and 9–10 years after the 9/11 WTC attacks	Questionnaires: PCL, PHQ-8, GAD-7	PTSD (12.9%); comorbidities: none (21.8%), depression (24.7%), anxiety (5.8%), both (47.7%)	PTSD with depression and anxiety, significantly more days with impaired mental health
Chen et al, 2020^35^	2029 police officers	Cross-sectional study without control group; 11–13 years after the 9/11 WTC attacks	Web-based survey	PTSD (9.3%) and (17.5%) PTSS (PCL-S)	Risk factors for PTSS: post-9/11 medical comorbidities, traumatic events
Chiu et al, 2011^36^	1915 retired male firefighters, mean age 47.5 years, 100% high school or higher, 83.3% married	Cross-sectional study without control group 4–7 years after the 9/11 WTC attacks	Structured diagnostic interview and questionnaires: DIS, PCL	6% PTSD	Retirement because of WTC-related disability ≥64%; 50% decrease in the likelihood of retirement because of a disability with each increase in age of 10 years
Cone et al, 2015^37^	2204 police officers, 86.6% male, mean age 38 years, 74.7% non-Hispanic White	Cross-sectional study without control group; 9–10 years after the 9/11 WTC attacks	Interview (not specified); questionnaires: PCL	PTSD (questionnaire: 11.0%); gender differences in PTSD: women 15.5%, men 10.3%	Correlation PTSD and lower social support; prevalence of PTSD increased with the number of life stressors in the past 12 months (waves 1 and 2 were not evaluated)
dePierro et al, 2021^38^	6777 police officers, 84% male, 39% non-Hispanic White (28% missing data), 19% Hispanic, 10% non-Hispanic Black, 99% high school or higher, 66% married or partnered	Cross-sectional study without control group; average 5 years after the 9/11 WTC attacks	Unstandardised interview and questionnaires: PCL-S, PHQ-9, CAGE	Less stigma in police officers compared with non-traditional responders (24.8 *v*. 41.2%); more stigma in police officers with psychiatric disorders compared with non-traditional responders with psychiatric disorders (17.9 *v*. 9.1%)	Mental disorder (17.4%); ‘I want to handle it myself’ was the most common reason for reluctance to seek help
Diab et al, 2020^39^	8881 police officers, 84.1% male, mean age 42.6 years, 55.2% non-Hispanic White, 25.6% Hispanic, 13.3% non-Hispanic Black, 98.9% more than high school education, 62.7% married or partnered	Cross-sectional study without control group; average 6.5 years after the 9/11 WTC attacks	Interview and questionnaires: PCL-S, PHQ-9, CAGE, SDS	20.6% need for mental healthcare (first annual health-monitoring visit with the WTC Health Program)	‘factors [ … ] associated with any perceived mental health service need were older age, female gender, diagnosis of depression/anxiety/PTSD before 9/11, positive screens for WTC-related PTSD, depression, and alcohol use problems, count of somatic diagnoses, and higher SDS score and more life stressors post 9/11’
Dowling et al, 2006^40^	28 232 notes from consultations of police officers	Retrospective cross-sectional study without control group 1–2 years after the 9/11 WTC attacks	Unstructured interview by paraprofessionals	Behavioural problems (34%), emotional problems (56%), physical problems (51%), cognitive problems (27%)	Evidence ‘that peer officers can be used effectively to assist police officers with post-disaster stress’
Feder et al 2016^41^	1874 police officers, 85.4% male, mean age 41.7 years, 68.1% non-Hispanic White, 20.0% Hispanic, 9.4% non-Hispanic Black, 83.9% more than high school education, 73.9% married or partnered	Longitudinal study without control group; 3, 6, 8 and 12 years after the 9/11 WTC attacks	Questionnaires: PCL-S health monitoring visit with the WTC Health Program	Four trajectories for PTSD symptoms: no/low symptoms (76.1%), worsening (12.1%), improvement (7.5%) and chronic (4.4%)	‘Positive emotion focused coping as well as current treatment were associated with the no/low symptom trajectory’
Gabriel et al, 2007^42^	153 police officers (46% of the total sample), 93% male, mean age 36.4 years, 98% Spain as country of origin, 57.5% primary school	Cross-sectional study without control group; 5–12 weeks after the 11 March 2004 terrorist attacks in Madrid	Questionnaires and interviews: DTS, MINI	Any mental disorder (3.9%), PTSD (1.3%), major depression (1.3%), agoraphobia (0.7%), GAD (0.7%), panic disorder (0.7%)	‘Intrusive ideas were the most frequent symptom’ of PTSD; comorbid mental disorders (2%)
Kotov et al, 2015^43^	8466 police officers, 85.3% male, mean age 40.9 years	Cross-sectional study without control group; average of 4 years after the 9/11 WTC attacks and 2.5 years after first assessment	PCL	PTSD 1-month prevalence: wave 1 (6.3%), wave 2 (8.2%)	Same sample as Pietrzak et al, 2012,^51^ with other questionnaire
Litcher-Kelly et al, 2014^44^	4035 police officers, 85.3% male, mean age 41.2 years, 67.8% non-Hispanic White, 20.3% Hispanic, non-Hispanic Black 9.8%, 84.0% more than high school education, 73.7% married or partnered	Longitudinal study without control group; 3 and 6 years after the 9/11 WTC attacks	Questionnaires: PCL-S	Psychological distress 3 years after 9/11 WTC predicted gastrointestinal symptoms 6 years after 9/11	‘Number of psychological distress symptoms 3–6 years after 9/11 WTC, predict the symptoms of the upper GI [gastrointestinal] 6 years after 9/11 WTC’
Luft et al, 2012^45^	8508 police officers, 85% male, mean age 40.8 years	Cross-sectional study without control group; 4 years after the 9/11 WTC attacks	Questionnaires: PCL	PTSD (5.9%)	No significant correlation between gender and PTSD (*r* = 0.05)
Mazza et al, 2012^46^	20 military police officers with PTSD, mean age 41.1 years	Cross-sectional study with control group; unspecified period after the terrorist attack in April 2006 in An-Nasiriyah, Iraq	Questionnaires and interviews: MSS, DTS, CAPS (DX), Strange Stories Test, Eyes Task, Emotion Attribution Task, Empathy Questionnaire	Only exposed personnel with PTSD were included (67.7%), and were compared with unaffected colleagues (33.3%)	Significantly less emotion recognition and empathy in affected personnel, problems in interpreting other people's mental states and emotions expressed by other individuals by only observing the ocular region
McCaslin et al, 2006^47^	166 police officers, 80.7% male, mean age 37.0 years, 52.8% non-Hispanic White, 17.8% Hispanic, 22.7% African American, 100% high school or higher, 59.3% married	Cross-sectional study without control group; short time after the 9/11 WTC attacks	Questionnaires and interviews: WEI, SOS, CAPS, PDEQ, PDI, LSC-R, TAS-20, PCL-C	Correlation with alexithymia and CAPS score (*r* = 0.35, *P* < 0.01)	Best predictors for PTSD symptoms were lack of social desirability, alexithymia, work environment stress, lack of social support, peritraumatic distress and peritraumatic dissociation
Motreff et al, 2020^21^	95 police officers, 203 firefighters, 229 health professionals, 134 NGO personnel; 62.0% male, mean age 37.5 years, 91.4% high school or higher	Cross-sectional study without control group; 8–12 months after the 13 November 2015 terrorist attacks in Paris	Questionnaires: WEI	Spatial and timely proximity to event (on unsecured crime scenes) increased PTSD risk (odds ratio 7.26); PTSD to PTSS prevalence: firefighters (3.4–15.7%), health professionals (4.4–10.4%), NGO personnel (4.5–19.4%), police officers (9.5–23.2%)	Increased risk for PTSD: female gender (odds ratio 1.3), no specific training (odds ratio 4.88), critical life events 1 year before (odds ratio 1.7), social isolation (odds ratio 8.4)
Perrin et al, 2007^48^	3925 police officers, 3232 firefighters, 1741 emergency medical services personnel, 5438 NGO personnel	Cross-sectional study without control group; 2–3 years after the 9/11 WTC attacks	Questionnaires: PCL-C	PTSD prevalence: police (7.2%), firefighters (14.3%), emergency medical services personnel (14.1%), NGO workers (8.4%) (PCL)	Maintaining an injury was the top risk factor for PTSD among firefighters, emergency services personnel and NGO workers
Pietrzak et al, 2014^49^	4035 police officers, 85.3% male, mean age 41.2 years, 67.8% non-Hispanic White, 20.3% Hispanic, non-Hispanic Black 9.8%, 84.0% more than high school education, 73.7% married or partnered	Longitudinal study without control group; 3, 6 and 8 years after the 9/11 WTC attacks	Questionnaires and interviews (as part of the health monitoring visit with the WTC Health Program): PCL-S, DIS	Four trajectories for PTSD symptoms: no/low symptoms (77.8%), worsening (8.5%), improvement (8.4%) and chronic (5.3%)	Risk factors for the severe chronic trajectory: ‘exposed to human remains, somatic injury/illness when at worksite, known someone injured there, traumatic death of college, friend or family member’
Pietrzak et al, 2014^50^	4035 police officers, 85.3% male, mean age 41.2 years, 67.8% non-Hispanic White, 20.3% Hispanic, non-Hispanic Black 9.8%, 84.0% more than high school education, 73.7% married or partnered	Longitudinal study without control group; 3, 6 and 8 years after the 9/11 WTC attacks	Questionnaires and interviews (as part of the health monitoring visit with the WTC Health Program): PCL-S, DIS	Five stable symptom clusters best represent PTSD symptom dimensionality in police officers	
Pietrzak et al, 2012^51^	8466 police officers, 85.2% male, mean age 49 years, 48.3% non-Hispanic White, 32.7% Hispanic, non-Hispanic Black 8.7%, 99.6% high school or higher, 71.3% married or partnered	Cross-sectional study without control group; average of 4 years after the 9/11 WTC attacks	Questionnaires and interviews (as part of the health monitoring visit with the WTC Health Program): PCL-S, DIS, PHQ-9, PHQ, CAGE, SDS, GHQ	One month prevalence of PTSD (5.4%) and PTSS (15.4%)	Risk factors for PTSD and PTSS: ‘older age, total number of exposures, exposed to human remains, early use on site, caught in dust cloud, early involved in search/rescue, lost someone, known someone who suffered injury, number of stressors before and after incident’
Schwarzer et al, 2014^52^	2943 police officers, 86.0% male, 73.9% White, 99% high school or higher, 74.1% married	Longitudinal study without control group; 1–2 and 6–7 years after the 9/11 WTC attacks	Questionnaires and interviews: PCL-C	Proximity to event as predictor to post-traumatic stress response	Social integration as resilience factor to stress
Schwarzer et al, 2016^53^	2204 police officers, 86.6% male, mean age 38 years, 74.7% non-Hispanic White	Longitudinal study without control group; 1–2, 6–7 and 9–10 years after the 9/11 WTC attacks	Questionnaires and interviews: PCL-C, MSSS	Exposure was correlated with symptoms (PTSD and stress) over time	The maximum mean symptom values are in wave 2; symptoms were negatively related to emotional support in wave 3
Vandentorren et al, 2018^54^	44 medical rescue workers, 60 firefighters, 56 police officers; 69% male, median age 36 years, 97.2% high school or higher, 62% married or partnered	Longitudinal study without control group; 6 and 18 months after the terror attacks in January 2015 in Paris	Questionnaires and face-to-face structured interviews: STRS, PDEQ, Sheehan Scale, MINI, PCL-S, CGI	PTSD prevalence (3% clinical interview); 8%, PCL-S), minimum of one anxiety disorder (14%); unable to work at some point since the attacks (half year; 6%, interview); increase alcohol, tobacco or cannabis use (9%, interview)	‘51% benefited from psychological support provided by their own institution’; seeking non-psychological healthcare since attacks (20%), with over 20% of them seeing a relationship to the incidents
Wesemann et al, 2020^13^	24 firefighters, 10 police officers, 13 NGO workers, 13 psychosocial workers; 70.0% male, 1.7% transgender; mean age 40 years, 14.4 mean years of service	Longitudinal study with control group; 3–4 month and 2 years after the terror attack in Berlin Breitscheidplatz, 2016	Questionnaire: BSI	More paranoid ideation in female responders at both time points; not found in controls	‘Paranoid ideation increased significantly over time in exposed female personnel’ compared with controls and male personnel; confirmation study for the 2016 study
Wesemann et al, 2020^55^	24 firefighters, 10 police officers, 13 NGO workers, 13 psychosocial workers; 70.0% male, 28.3% female, 1.7% transgender; mean age 40 years, 14.4 mean years of service	Longitudinal study with control group; 3–4 month and 2 years after the terror attack in Berlin Breitscheidplatz, 2016	Questionnaire: WHOQOL-BREF, BSI	More hostility in police officers at both time points compared with controls; less environmental quality of life in firefighters at both time points compared with controls	Physical quality of life of the firefighters was no longer impaired 2 years after the incident; confirmation study for the 2016 study
Wesemann et al, 2018^56^	16 firefighters, 6 police officers, 6 psychosocial workers; 70% male, mean age 42.8 years, 97.3% high school or higher, 61.7% married or partnered	Cross-sectional study without control group; 3 months after the terror attack in Berlin Breitscheidplatz, 2016	Questionnaire: PHQ, WHOQOL-BREF, BSI	More stress and paranoid ideation in female responders; less environmental and physical quality of life in firefighters, more hostility in police officers	
Wisnivesky et al, 2011^57^	12 273 protective services or military, 86% male, median age 38 years, 57% non-Hispanic White, 31% Hispanic, non-Hispanic Black 11%, 99.6% high school or higher, 71.3% married or partnered	Longitudinal study without control group; yearly 1–9 years after the 9/11 WTC attacks	Questionnaires and interviews: PHQ, PCL-S,	Cumulative incidence of depression (7.0%), PTSD (9.3%) and panic disorder (8.4%)	Steady yearly increase in the incidence rates from year 1 to year 9 for depression (1.7 to 7.0%), PTSD (2.5 to 9.3%) and panic disorder (2.3 to 8.4%)
Wyka et al, 2020^58^	12 398 rescue/recovery forces not further specified (police officers, firefighters, emergency medical, technicians, sanitation workers and volunteers); 79.2% male, 45.1% aged >42 years, 80.3% non-Hispanic White, 44.4% college degree or higher	Longitudinal study without control group; 2–3, 5–6 and 9–10 years after the 9/11 WTC attacks	Questionnaires: PCL	PTSD wave 1 (19.6%), wave 2 (18.2%) and wave 3 (16.7%)	
Zvolensky et al, 2015^59^	8466 police officers, 85.3% male, mean age 37.1 years, 20.7% Hispanic, 20.6% non-Hispanic Black	Longitudinal study without control group; 1–9 years after the 9/11 WTC attacks and an average of 2.5 years after first assessment	Questionnaires: PCL, SDS	Greater initial disaster exposure lead to more PTSS and decreased overall functioning over time	‘Post-disaster life stressors moderated the negative effects. These effects were not found in non-traditional WTC-responders’
Zvolensky et al, 2015^60^	763 (ex-) smoking police officers, 78.9% male, mean age 37.3 years	Longitudinal study without control group; 1–9, years after the 9/11 WTC attacks and an average of 2.5 years after first assessment	Questionnaires and interviews: PCL-S	Higher levels of PTSS at wave 1 were associated with a decreased likelihood of smoking abstinence and with decreased smoking reduction at wave 2	‘Hyperarousal PTSD symptoms were predictive of decreased abstinence likelihood at wave 2’
Zvolensky et al, 2015^61^	8466 police officers, 5.3% male, mean age 37.1 years, 20.7% Hispanic, non-Hispanic Black 20.6%	Longitudinal study without control group; 1–9, years after the 9/11 WTC attacks and an average of 2.5 years after first assessment	Questionnaires and interviews: PCL-S, PHQ-9, SDS	Post-traumatic stress, depressive symptoms and overall functioning were stable over the follow-up period	Post-event job-loss, layoff or substantial loss of income (11.3%), change in living arrangements (17.3%), legal problems (4.0%), arrested (0.6%)

All studies have a naturalistic design (terrorist attack). WTC, World Trade Center; SF-8 Health Survey, Short-Form health-related quality of life; THQ, Trauma History Questionnaire; PDEQ, Peritraumatic Dissociative Experiences Questionnaire; ASD, acute stress disorder; PCL, PTSD Checklist; PTSD, post-traumatic stress disorder; PTSS, post-traumatic stress smptoms; PHQ-8, Patient Health Questionnaire-8 depression scale; GAD-7, Generalised Anxiety Disorder scale; PCL-S, PTSD Checklist-Specific Stressor version; DIS, Diagnostic Interview Schedule; PHQ-9, Patient Health Questionnaire-9 depression scale; CAGE, cut-annoyed-guilty-eye (alcohol); SDS, Sheehan Disability Scale; DTS, Davidson Trauma Scale; MINI, Mini International Neuropsychiatric Interview; GAD, generalised anxiety disorder; MSS, Mississippi Scale; CAPS, Clinically Administered PTSD Scale; WEI, Work Environment Inventory; SOS, Sources of Support Scale; PDI, Peritraumatic Distress Inventory; LSC-R, Life Stressor Checklist-Revised; TAS-20, Toronto Alexithymia Scale; PCL-C Posttraumatic Stress Checklist-Civilian Version; NGO, nongovernmental organisation; GHQ, General Health Questionnaire; MSSS, Modified Social Support Survey; STRS, Shortness of Breath, Tremulousness, Racing heart and Sweating scale; CGI, Clinical Global Impression questionnaire; BSI, Brief Symptom Inventory; WHOQOL-BREF, World Health Organisation Quality of Life.

### Risk-of-bias assessment

A major selection bias in these naturalistic studies is the involvement of the participants. To control for this, we checked the literature for recruitment strategies (e.g. participants who have come to an aid programme of any kind, evaluation of intervention strategies, etc.) or whether all persons in the total sample had the same chance of being included. This information was taken into account in the qualitative evaluation of the results. In addition, we checked whether the same personnel were included in different studies. When evaluating the results, possible overlaps of personnel participating in multiple studies were taken into account. The risk assessment was carried out by one author according to the Quality Assessment Tool for Quantitative Studies, from the Effective Public Health Practice Project Quality Assessment Tool,^62^ and assessed by a second author. Disagreements were resolved across the study group. The ‘study design’ component was reassessed, as almost all studies were rated as ‘weak’ because of the naturalistic nature of these studies.

This assessment was done in accordance with the suggestions of the Evidence-based Practice Center of the Agency for Healthcare Research and Quality (AHRQ). It states that the assessment should take into account various sources of design bias rather than assessing individual studies for the study design itself. This would put studies at a disadvantage if they were downgraded by the risk of bias solely based on the study design.^63^ This method was also chosen based on the Newcastle-Ottawa Scale to assess the quality of non-randomised studies.^64^ The Newcastle-Ottawa Scale is a not yet established assessment tool for cohort studies. For this reason, we have retained the ‘Quality Assessment Tool for Quantitative Studies’ criteria for this adaptation. According to the AHRQ guideline, we upgraded the study design from weak to moderate if they had large, consistent effects.

Of the 33 included studies, 11 were rated as strong, 19 as moderate and three as weak, as shown in Table 2.

**Table 2:** Quality assessment of the included studies according to the Quality Assessment Tool for Quantitative Studies

Reference	Name	A	B	U/D	C	D	E	F	G	GN
31	Biggs et al, 2010	2	3	3	2		1	./.	2	2
32	Bowler et al, 2010	2	3	2	1		1	./.	2	1
33	Bowler et al, 2012	2	3	2	1		1	2	2	2
34	Bowler et al, 2016	2	3	2	1		1	3	3	2
35	Chen et al, 2020	3	3	2	2		2	./.	3	2
36	Chiu et al, 2011	2	3	2	1		1	./.	2	1
37	Cone et al, 2015	3	3	2	1		1	./.	3	2
38	dePierro et al, 2021	2	3	2	2		2	./.	2	1
39	Diab et al, 2020	2	3	2	2		1	./.	2	1
40	Dowling et al, 2006	3	3	3	2		3	./.	3	3
41	Feder et al, 2016	2	3	2	1		1	2	2	1
42	Gabriel et al, 2007	3	3	2	1		1	./.	3	2
43	Kotov et al, 2015	2	3	2	1		1	./.	2	2
44	Litcher-Kelly et al, 2014	2	3	3	2		1	2	2	2
45	Luft et al, 2012	2	3	3	2		1	./.	2	2
46	Mazza et al, 2012	3	2	2	3		1	./.	3	3
47	McCaslin et al, 2006	3	3	3	1		1	./.	3	3
21	Motreff et al, 2020	2	3	2	1		1	./.	2	1
48	Perrin et al, 2007	2	3	2	1		1	./.	2	1
49	Pietrzak et al, 2014	2	3	2	2		1	3	3	2
50	Pietrzak et al, 2014	3	3	2	2		1	3	3	2
51	Pietrzak et al, 2012	2	3	2	2		1	2	2	1
52	Schwarzer et al, 2014	2	3	2	1		1	2	2	1
53	Schwarzer et al, 2016	2	3	3	1		1	2	2	2
54	Vandentorren et al, 2018	2	3	2	1		1	./.	2	1
13	Wesemann et al, 2020	3	2	2	1		1	2	2	2
55	Wesemann et al, 2020	3	2	2	1		1	2	2	2
56	Wesemann et al, 2018	3	3	2	1		1	./.	3	2
57	Wisnivesky et al, 2011	1	3	3	1		1	2	2	2
58	Wyka et al, 2020	1	3	3	1		1	2	2	2
59	Zvolensky et al, 2015	1	3	2	1		1	2	2	1
60	Zvolensky et al, 2015	2	3	3	1		1	2	2	2
61	Zvolensky et al, 2015	1	3	3	1		1	2	2	2

A, selection bias; B, study design; U/D, upgrade/downgrade study design; C, confounders; D, blinding (not applicable); E, data collection; F, drop-out; ./., no data available or not applicable due to study design; G, global assessment; GN, global assessment after upgrade/downgrade (1, strong; 2, moderate; 3, weak); GN: 1 = no weak rating; 2 = one weak rating; 3 = two or more weak ratings.

### Strategy for data synthesis

Because of the small number of included studies, no minimum was specified for defining the synthesis. A bias in the selection of recruitment (as described above) was taken into account for the final synthesis. Otherwise, all prevalence data on mental disorders were given for the individual occupational groups. The synthesis was carried out according to the ‘Guidance on the Conduct of Narrative Synthesis in Systematic Reviews’.^65^ Finally, all first authors with more than two of the included and analysed studies were consulted to verify the validity of the synthesis, which was modified accordingly.

## Results

### Data collection

The initial and further literature search resulted in 56 papers. Eleven were excluded because they were not related to rescue workers, three did not report their own data, two had no focus on mental health or were a meta-analysis, one was excluded because it was not in the English language and one was excluded because the results were not broken down by professional groups. As a result, 33 full-text articles were included for primary analysis.

### Terrorist attacks

**Fig. 1:**
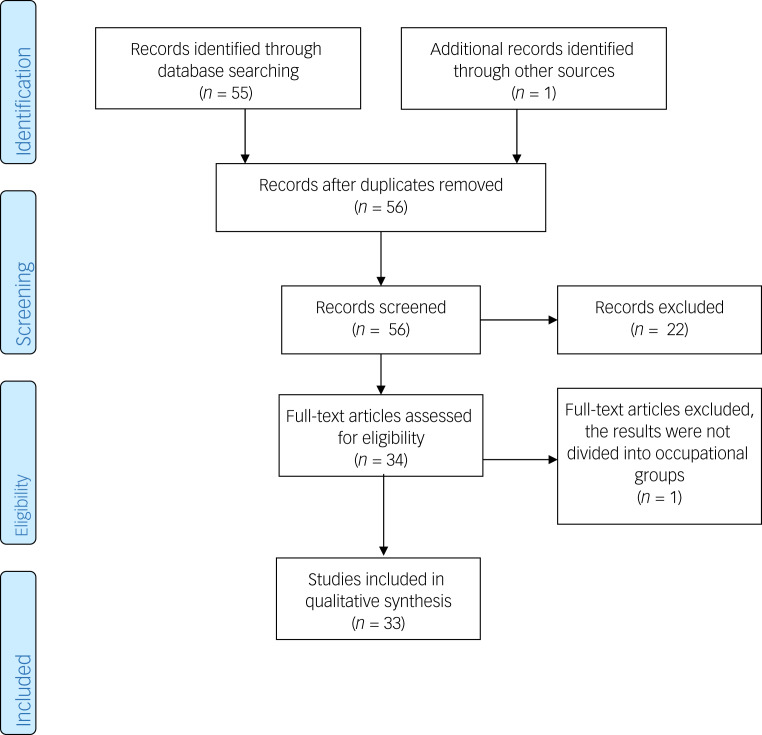
Preferred Reporting Items for Systematic Reviews and Meta-Analyses (PRISMA) flow diagram.

The most comprehensive work concerned the terrorist attacks on the World Trade Center in New York, USA on 11 September 2001 (hereafter referred to as 9/11 WTC; *n* = 25 publications); followed by the terrorist attack on the Christmas market at Breitscheidplatz in Berlin, Germany on 19 December 2016 (*n* = 3); the terrorist attacks in Paris, France in 2015 (*n* = 2); the terrorist attack in Madrid, Spain on 11 March 2016 (*n* = 1) and the terrorist attack on Nasiriyah, Iraq in April 2006 (*n* = 1). The inclusion process is presented in Fig. 1.

### Descriptive statistics of the studies

The publication dates range from 2006 to 2021, with mean and median years between 2011 and 2015. Almost all studies included police officers (*n* = 30), followed by firefighters (*n* = 6), paramedics and NGOs (each *n* = 5), psychosocial workers (*n* = 3) and search and rescue workers (*n* = 2). There is a wide range of participants of each included study, ranging from 28 to 28 232 participants. The data was collected between 2 weeks and 20 years after the incidents. The exact time of data collection was not given in two studies. Almost half of the studies were cross-sectional (*n* = 17) or longitudinal (*n* = 16). Only three studies included a control group from the same occupational group that was not used in the incident.

### Prevalence rates within the first 3 months after the terrorist attack

An acute stress disorder (ASD) was identified in 14.6% of the emergency services (including 90 medical personnel, police officers and firefighters) 2–3 weeks after the 9/11 WTC attacks. Depression (results from questionnaires) was reported in 25.8% of the cases, high psychological impairment in 33.3% and at least one peritraumatic dissociation symptom in 83.3%. The comorbidity of ASD and depression was 10.9%. The prevalence rate of ASD was highest among tobacco users, at 33.3–37.9%.^32^ At 5–12 weeks after the 11 March 2004 terrorist attacks in Madrid, the prevalence of any mental disorder was 3.9% for 153 police officers, including 1.3% with PTSD (with intrusive ideas as the most common symptom), 1.3% with major depression, 0.7% with agoraphobia, 0.7% with generalised anxiety disorder and 0.7% with panic disorders. Furthermore, 51% of the police officers with a mental disorder suffered from another comorbid mental disorder.^42^

### Prevalence rates within the first year after the terrorist attack

At 6–9 months after the January 2015 Paris terrorist attacks, the PTSD prevalence among police officers was between 3% (interview) and 8% (questionnaire). Other police officers reported at least one anxiety disorder (14%), an inability to work after the attacks (6%) and an increase in alcohol, tobacco or cannabis use (9%).^54^ At 8–12 months after the attacks, the PTSD and PTSS rate was 3.4 and 15.7% in firefighters (*n* = 203), 4.4 and 10.4% in health professionals (*n* = 229), 4.5 and 19.4% in personnel of NGOs (*n* = 134) and 9.5 and 23.2% in police officers (*n* = 95). The spatial proximity to the unsecured crime scenes increased the risk of having PTSD (odds ratio 7.26).^21^

### Prevalence rates during the first and second years after the terrorist attack

At 1–2 years after the 9/11 WTC attacks, 28 232 notes from consultations of police officers were evaluated. The notes evaluated were from unstructured interviews done by paraprofessionals. The prevalence of behavioural problems (e.g. hypervigilance, isolation, drug use or violent behaviour) was found in 34% of police officers, emotional problems (e.g. anger, sadness, anxiety or hopelessness) in 56%, physical problems (e.g. fatigue, headaches, stomach problems or chronic agitation) in 51% and cognitive problems (e.g. concentration, intrusive thoughts, decision-making or preoccupation with death) in 27%.^41^

### Prevalence rates during the second and third years after the terrorist attack

At 2–3 years after the 9/11 WTC attacks, the prevalence rate of PTSD (results from questionnaires) in 4017 police officers was 7.8%.^32^ Emotional numbness was found in 6.7%.^33^ Another study referring to the same event found PTSD prevalence rates (results from questionnaires) for 3925 police officers (7.2%), 3232 firefighters (14.3%), 1741 emergency medical service (14.1%) and 5438 personnel from NGOs (8.4%).^48^

### Prevalence rates during the fourth and fifth years after the terrorist attack

At 4 years after the 9/11 WTC attacks, 8508 police officers were screened for PTSD, and the prevalence (results from questionnaires) was 5.9%.^45^ In the second wave of the study,^33^ 5–6 years after the 9/11 WTC attacks, 2940 police officers were screened and the prevalence rate climbed significantly, to 16.5%. Emotional numbness also climbed from 6.7 to 15.6%.^34^ A total of 8466 police officers were screened an average of 4 years after the 9/11 WTC attacks. The 1-month prevalence (interview) of PTSD was 5.4% and prevalence of PTSS was 15.4%.^51^ Among 1915 retired firefighters 4–7 years after the 9/11 WTC attacks, the prevalence rate of PTSD was similarly high, with 6% affected (interview). Retirement was the result of a disability related to the attacks in 64% of respondents.^34^ Five years after the 9/11 WTC attacks, 6777 police officers were subjected to non-standardised interviews. The prevalence of any kind of mental disorder was 17.4%.^38^

### Prevalence rates 6–10 years after the terrorist attack

An average of 6.5 years after the 9/11WTC attacks, 8881 police officers were interviewed and completed questionnaires as part of the first annual health surveillance visit with the WTC Health Program; 20.6% of the participants required mental healthcare.^39^ Nine to 10 years after the attacks, the prevalence rate of PTSD (results from questionnaires) for 2204 police officers was 11.0%.^37^ Another study 9–10 years after the attacks reported a 12.9% PTSD prevalence (results from questionnaires) in police officers. The comorbidity categories of those affected included depression (24.7%), anxiety (5.8%), both (47.7%) and none (21.8%).^34^

### Prevalence rates more than 10 years after the terrorist attack

In a web-based survey 11–13 years after the 9/11 WTC attacks, including 2029 police officers, the incidence rate of PTSD (results from questionnaires) was 9.3% and the incidence rate for PTSS was 17.5%.^35^

### Gender and occupational differences

Three months after the terror attack at Berlin's Breitscheidplatz in 2016, there was more reported stress and paranoid ideation in female responders, less environmental and physical quality of life in firefighters and more hostility in police officers.^56^ A confirmation study 3–4 months and 2 years after the terror attack replicated more paranoid ideation in female responders at both time points. Paranoid ideation increased significantly over time in exposed female personnel compared with controls.^13^ Also, more hostility was displayed in police officers at both time points, as well as less environmental quality of life in firefighters at both time points, compared with controls. Physical quality of life of the firefighters was no longer impaired 2 years after the incident.^55^ At 2–3 years after the 9/11 WTC attacks, gender differences in the prevalence rates of PTSD (results from questionnaires) in 4017 police officers were found, with 13.9% in women and 7.4% in men.^32^ Four years after the 9/11 WTC attacks, no significant correlation between gender and PTSD (*r* = 0.05) was found in 8508 police officers.^45^ In the second wave of this study, 2940 police officers were surveyed 5–6 years after the 9/11 WTC attacks. No gender-specific differences were found in the PTSD prevalence. Nevertheless, there were more intrusive symptoms in women.^33^ At 9–10 years after the 9/11 WTC attacks, in the remaining 2204 police officers, the gender differences in PTSD became prevalent again, with 15.5% in women and 10.3% in men.^37^

### Risk factors for mental disorders

Risk factors for PTSD were found 8–12 months after the November 2015 terrorist attacks in Paris. These risk factors include female gender (odds ratio 1.3), no training on psychological risks of this type of event (odds ratio 4.88), critical life events 1 year before the incident (odds ratio 1.7) and social isolation (odds ratio 8.4).^21^ Shortly after the 9/11 WTC attacks, there was a correlation between alexithymia and PTSD (*r* = 0.35; *P* < 0.001) in 166 exposed police officers. Additionally, psychological distress 3 years after the 9/11 WTC attacks predicted gastrointestinal symptoms 6 years after the incident.^44^

For those with PTSS, the best predictors were lack of social desirability, alexithymia, work environment stress, lack of social support, peritraumatic distress and peritraumatic dissociation.^47^ General risk factors in 4017 police officers 2–3 years after the 9/11 WTC attacks were event-related injury and older age.

There were several gender-related risk factors identified. Special risk factors for men were proximity to the event and Hispanic ethnicity. In women, risk factors included witnessing the horrific event and an education level below a college degree.^32^

Additionally, risk factors were identified and split by personnel. Maintaining an injury was the top risk factor for PTSD among firefighters, emergency services and NGO workers 2–3 years after the 9/11 WTC attacks.^48^ In 1915 retired firefighters 4–7 years after the 9/11 WTC attacks, there was a 50% decrease in the likelihood of retirement because of a disability with each increase in age of 10 years.^36^

In 8466 police officers an average of 4 years after the 9/11 WTC attacks, the risk factors for PTSD and PTSS were older age, the total number of exposures, exposure to human remains, caught in a dust cloud, involved in the early stages of search/rescue, known someone who had died in the attacks, known someone who suffered an injury in the attacks, and the number of stressors before and after the incident.^51^

In a web-based survey of 2029 police officers 11–13 years after the 9/11 WTC attacks, risk factors for PTSS were post-event medical comorbidities and other traumatic events.^35^ An average of 6.5 years after the 9/11 WTC attacks, risk factors for any perceived mental health service need were assessed in 8881 police officers. Risk factors included old age; female gender; a diagnosis of depression, anxiety and/or PTSD before the attacks; positive screens for PTSD related to the attacks; alcohol misuse; several somatic diagnoses; functional impairment in work and more life stressors since the attacks.^39^

In a longitudinal study with measurements 3, 6 and 8 years after the 9/11 WTC attacks, 4035 police officers were assessed. Risk factors for the severe chronic trajectory were exposure to human remains, on-site somatic injuries/illnesses, acquaintances with injuries and the traumatic death of a colleague, friend or family member.^49^ At 9–10 years after the 9/11 WTC attacks, low social support was a risk factor for PTSD in 2204 police officers. The prevalence of PTSD increased with the number of life stressors that occurred within the past 12 months.^37^

### Trajectories and temporal course

In a longitudinal study with measurements 3, 6 and 8 years after the 9/11 WTC attacks, 4035 police officers were included. Four trajectories for PTSD symptoms were found, including no/low symptoms (77.8%), worsening symptoms (8.5%), symptom improvement (8.4%) and a chronic (5.3%) trajectory.^49^ The same study with the remaining 1874 police officers and an additional assessment 12 years after the 9/11 WTC attacks was performed. Four trajectories for PTSD symptoms were found, which included no/low symptoms (76.1%), worsening symptoms (12.1%), symptom improvement (7.5%), and a chronic (4.4%) trajectory.^41^ Another longitudinal study 1–2, 6–7 and 9–10 years after the 9/11 WTC attacks included 2204 police officers. The exposure event was correlated with PTSD and stress symptoms over time. The peak symptom trajectory was found in wave 2. The symptoms were negatively related to emotional support in wave 3.^53^ In a yearly study from year 1 to year 9 after the 9/11 WTC attacks, 12 273 protective service or military personnel were included. The cumulative incidence of depression was 7.0%, cumulative incidence of PTSD was 9.3% and cumulative incidence of panic disorders was 8.4%. The study found a steady yearly increase in the incidence rates from year 1 to year 9. Depression rose from 1.7 to 7.0%, PTSD rose from 2.5 to 9.3% and panic disorders rose from 2.3 to 8.4%.^57^ Another longitudinal study with 12 398 rescue workers at 2–3, 5–6 and 9–10 years after the 9/11 WTC attacks showed the opposite results. The PTSD prevalence (results from questionnaires) decreased from wave 1 (19.6%) to wave 2 (18.2%) and in wave 3 (16.7%). The sample included police officers, firefighters, paramedics, technicians, plumbers and volunteers. However, there is no differentiation between these subgroups.^58^ Another year 1 to year 9 longitudinal study after the 9/11 WTC attacks included 8466 police officers. A greater initial disaster exposure led to more PTSS and a decrease in overall functioning over time. Post-disaster life stressors amplified these negative effects. These effects were not found in non-traditional WTC-responders.^59^ In this study, PTSD, depressive symptoms and overall functioning were stable over the follow-up period.^61^ In the same study, a subgroup analysis was done for 763 police officers who were ex-smokers. Higher initial PTSS were associated with a decreased likelihood of smoking abstinence and decreased smoking reduction. Hyperarousal also indicated a decreased likelihood of abstinence over time.^60^

Kotov et al found an increase in the 1-month prevalence rate of PTSD (results from questionnaires) in 8466 police officers from 6.3% of responders an average of 4 years after the 9/11 WTC attacks. This prevalence rate increased to 8.2% an average of 2.5 years after the first assessment.^43^

A longitudinal study investigated 2204 police officers 1–2, 6–7 and 9–10 years after the 9/11 WTC attacks. Exposure was correlated with symptoms of PTSD and stress over time.^53^

### Resilience factors and helpful support

After the 9/11 WTC attacks, there was evidence that peer officers can be used effectively to assist police officers with post-disaster stress.^40^ A similar result emerged after the Paris terrorist attacks in 2015. More than half of the rescue workers affected (51%) benefited from their institution's psychological help.^54^ In a longitudinal study with measurements 3, 6, 8 and 12 years after the 9/11 WTC attacks, 1874 police officers were sampled. Positive emotional coping and current treatment were associated with the no/low symptom trajectory for PTSD.^41^ In a longitudinal study with 2943 police officers 1–2 and 6–7 years after the 9/11 WTC attacks, social integration was identified as a resilience factor to PTSS.^52^

### Stigma

About 5 years after the 9/11 WTC attacks, 6777 police officers were subjected to non-standardised interviews. Results showed there was less stigma surrounding psychiatric disorders in police officers compared with non-traditional responders (24.8 *v*. 41.2%). Nevertheless, it was found that police officers with psychiatric disorders have greater self-stigma than non-traditional responders with psychiatric disorders (17.9 *v*. 9.1%). ‘Trying to deal with it yourself’ was the most common reason for the reluctance to seek help.^38^

### Problems in daily life after the events

In the course of the next 9 years after the 9/11 WTC attacks, 8466 police officers were asked about critical post-disaster life events: job loss, layoff or substantial loss of income was reported in 11.3%; changes in living situation were reported in 17.3%; legal problems were reported in 4.0% and arrest was reported in 0.6%.^61^ Military police officers with PTSD after a terrorist attack in Iraq showed significantly less emotional recognition and empathy, and problems interpreting other people's mental states and expressed emotions.^46^

### Test instruments

In a longitudinal study with measurements 3, 6 and 8 years after the 9/11 WTC attacks, 4035 police officers were included. In both the interviews and questionnaires, five stable symptom clusters best represented the PTSD symptom dimensionality in police officers. The symptom clusters included re-experiencing (e.g. intrusive thoughts), avoidance (e.g. avoidance of thoughts), numbing (e.g. trauma-related amnesia), dysphoric arousal (e.g. irritability) and anxious arousal (e.g. overly alert).^50^

Most studies used questionnaires rather than interviews. The most common instrument was the PTSD Checklist (PCL) with its variants. Comparing the PCL-S (specific version) and PCL-C (civilian version) between the same 8466 police officers, the prevalence rate for PTSD ranged from 5.4^51^ to 6.3%.^43^

## Discussion

The prevalence rates of mental disorders after a terrorist attack vary widely. For PTSD, the range is 1.3–16.5%, for major depression, it is 1.3–25.8% and for specific anxiety disorders, it is 0.7–14%.^33,42,54^ This also applies to the prevalence rate in the same occupational group after the same incident. The largest discrepancy exists between questionnaires and clinical interviews, with higher rates of reported mental disorders in the questionnaires.^54^ Since a psychiatric interview is the gold standard for diagnosis, the questionnaire results are believed to overestimate prevalence rates. This is attributed to the specificity. The lower the specificity of the questionnaires, the higher the overestimation. In the case of questionnaire results being utilised in future research, this should always be described in the limitations and, if possible, the positive predictive value should be given.

From an academic point of view, it would be desirable to have a special set of instruments for evaluating such extraordinary events. This would make the studies more comparable. For this purpose, we recommend any type of evaluated and standardised clinical interview that relates to the current versions of the ICD-11 or DSM-5. The interviews should be conducted by trained and specialised staff, and should cover the most common mental disorders. At a minimum, it should include depressive episodes, anxiety disorders, PTSD, psychosomatic disorders and substance misuse disorders. If such an interview cannot be conducted, validated questionnaires with high sensitivity and specificity are recommended.

The highest prevalence rates were found after the 9/11 WTC attacks. This is attributed to the particular nature of the terrorist attack: it comprised multiple incidents and there was a significant length of time after the attacks until the buildings collapsed. The likelihood of developing dissociative symptoms during this time is higher.^32^ It is, therefore, not surprising that 2–3 after this attack, 83.3% of the disaster relief workers reported at least one peritraumatic dissociation symptom.^31^

Given the wide variation of prevalence rates in studies covering the same occupational groups, it seems difficult to compare studies covering different occupational groups. To be able to make conclusive statements, only studies that include different professional groups and differentiated accordingly were considered. Three months after the Berlin terrorist attack in 2016, deployed firefighters, health professionals, NGO personnel and police officers were compared. Firefighters showed the least environmental and physical quality of life, whereas police officers showed more hostility.^56^ Aside from the physical quality of life, these results were replicated 2 years after this terrorist attack and were not found in the non-deployed comparison group.^55^ In the first year after the January 2015 Paris terror attacks, the PTSD point prevalence rate was 3.4% in firefighters, 4.4% in health professionals, 4.5% in NGO personnel and 9.5% in police officers.^54^ During the second and third years after the 9/11 WTC attacks, this order was reversed. The point prevalence for PTSD (results from questionnaires) was 14.3% for firefighters, 14.1% for health professionals, 8.4% for NGO personnel and 7.2% for police officers.^48^

These results support the necessity to distinguish between the specific occupational groups. However, they also refer to the importance of the specific task of the emergency services personnel. For example, police officers who searched for perpetrators after the Paris attacks had the highest prevalence rates for PTSD compared with other occupations, whereas police officers who were more likely to conduct evacuations after the 9/11 WTC attacks had the lowest PTSD rates. This task-related hypothesis would also explain the occupational differences identified after the Berlin terrorist attack. Although firefighters concentrated on first aid and risk reduction at the destroyed Christmas market, police officers secured the crime scene from further attacks and searched for the perpetrator. A focus on destruction would then explain a reduction in environmental quality of life, whereas a focus on perpetrators would explain an increase in hostility.

A more consistent picture emerges when it comes to gender differences. If differences are found, these are at the expense of female emergency services personnel with more mental health symptoms^13,33,56^ or higher prevalence rates of mental disorders.^32,37^ The more frequent harassment of minorities in male-dominated professions was given as an explanation for these results.^13^ However, since such differences also occur among non-underrepresented female employees, other factors seem to play a more important role. Risk factors identified included lower education and witnessing horror.^32^ A combination of situational factors (e.g. horror experiences) and personal factors (e.g. lower education) may account for these differences. In addition to age, ethnicity and level of education, it is therefore advisable to include marital status, relationships with colleagues and supervisors, and general job satisfaction in future studies. In addition to the duration and proximity to the event, the situation-specific aspects also include personal risk perception, preparation for the event, confidence in action, clarity of tasks on-site, quality of equipment, professional experience and media reports on the organisation's emergency services personnel (positive/negative).

However, screening for mental health symptoms (after a terroristic event) remains necessary to prevent or treat mental disorders. This could be done in a two-stage strategy, with an initial questionnaire and an additional interview for at-risk employees. Screening should always be voluntary to avoid simulation and dissimulation. The screening should also be repeated at regular intervals. The data from this review is inconsistent as to whether mental health problems increase or decrease over time. This is likely because of the different trajectories found in the longitudinal studies.^41,49^

This screening strategy could help reduce the high prevalence rate of mental disorders. The focus of the identified studies was clearly on PTSD. However, PTSD is just one of several mental disorders that can be exacerbated after a terroristic event. Depression, anxiety disorders, substance use disorders and psychosomatic disorders should also be considered. Most of the studies in which measurements were taken to detect these disorders showed increased prevalence rates.^34^ Screening for life events before and after the incidents also appeared to be helpful.^21,44^ These catastrophic events tend to increase vulnerability rather than resilience or post-traumatic growth.^51,59^ Another critical incident following the first exposure could more quickly lead to mental health problems.^37^

The main risk factors identified in the studies were female gender,^21,39^ no training on psychological risks of this type of event,^21,44^ social isolation,^37,47^ work environment stress,^32,47^ peritraumatic distress and peritraumatic dissociation,^39,47^ losing someone or maintaining an injury,^48,49^ and older age.^32,36^ This knowledge could be used in special training courses for emergency personnel dealing with catastrophic events. Each occupational group could implement prevention strategies for these risk factors. Failure to prepare for this type of emergency response resulted in an odds ratio of 4.88 for mental disorders.^21^ The same applies to post-deployment strategies. Semi-professional psychological crisis interventions should focus on all types of mental symptoms. Knowing about specific risk factors could help to make them more target-group-specific. Institutional peer support has been shown to be effective after the 9/11 WTC attacks,^40^ and is probably the most appropriate way to deal with such huge incidents. In addition, destigmatisation programmes showed evidence of a reduction in psychological stress^66,67^ and the need to implement them.^68^ This could be another important tool for mental health prevention, as the perceived stigma of the emergency workers involved is comparatively high.^38^

There are some study limitations to report. Results may be limited because only two databases were used, only English-language literature was considered, results for female emergency services are limited because of the high proportion of males in emergency services personnel jobs, and the search strategy may have missed other events. Finally, the sample size for police officers was predominant out of all of the occupational groups identified.

In conclusion, given the trajectories and time course of mental disorders,^41,49,53^ voluntary routine screening over an extended period of at least 7 years after the incident is strongly recommended. Otherwise, a notable part of the emergency services personnel could easily be missed in the ‘deterioration curve’ if mental symptoms appear later. If this screening is carried out with questionnaires, a clinical interview could confirm the emergency services personnel with a mental disorder diagnosis. This can minimise the rate of false-positive results.

Additionally, research using only questionnaires should always report the positive predictive value of the questionnaires. For questionnaires with low specificity that search for rare diseases, the rate of false positives can be many times higher than the rate of true positives.

The stigma associated with mental disorders remains a major barrier for emergency services personnel in seeking professional help.^38^ Destigmatisation programmes should therefore be implemented or further expanded in all relevant institutions. There are already a number of established options.^66–68^ If mental damage caused by deployment is recognised as an accident at work and mental disorders are assessed by the employer as an occupational risk, this could have a positive effect on recovery.

The identified risk factors can be taken more into consideration when coordinating primary and secondary prevention. As a result, the programmes will be tailored more specifically to the needs of the organisations and those affected. Very general programmes, or even the lack thereof, are associated with poorer mental health outcomes.^40,41,54^

Further research on this topic is needed to better understand the interaction of risk factors for the development of mental disorders. A distinction must be made between situational factors such as proximity to the event, personal factors such as gender, a combination of both situational and personal factors such as personal risk perception, and occupational factors such as specific tasks and organisational factors such as training. Additionally, a distinguishment must be made between intentionally caused major events such as terrorist attacks and unintentionally caused events such as natural disasters, including the severity and duration of the incidents to effectively compare the impact of both types of events.

## Data Availability

Data availability is not applicable to this article as no new data were created or analysed in this study.
